# Serum C3 and Renal Outcome in Patients with Primary Focal Segmental Glomerulosclerosis

**DOI:** 10.1038/s41598-017-03344-1

**Published:** 2017-06-22

**Authors:** Jian Liu, Jingyuan Xie, Xiaoyan Zhang, Jun Tong, Xu Hao, Hong Ren, Weiming Wang, Nan Chen

**Affiliations:** 1 Institute of Nephrology, Shanghai Jiao Tong University School of Medicine, Shanghai, China; 2 Department of Nephrology, RuiJin Hospital, Shanghai Jiao Tong University, School of Medicine, 200025 Shanghai, P.R. China

## Abstract

The role of complement (C) in the pathogenesis or progression of focal segmental glomerulosclerosis (FSGS) is uncertain. The present study assessed the relationship between serum C3, the baseline characteristics, and the progression of FSGS in the cohort and identified the clinical implications of serum C3 levels in patients with FSGS. Compared to the patients with C3 ≥ 85 mg/dL (N = 474), those with C3 < 85 mg/dL (N = 117) presented a higher level of serum creatinine, lower levels of eGFR, hemoglobin, proteinuria, triglyceride, cholesterol, IgA, as well as, severe tubulointerstitial injury (TI). Of the 221 patients with a mean follow-up of 53.3 months, the risk of reaching end-stage renal disease (ESRD) was significantly higher in patients with low serum C3 level (p < 0.001). An additional 40 patients with primary FSGS revealed a significant correlation between MAC and AP (p = 0.003), MAC and serum C3 (p = 0.018), and AP and serum C3 (p = 0.028). Compared to patients with none-to-mild TI, those with moderate-to-severe TI exhibited a lower level of serum C3 and AP, and a higher level of serum MAC. In conclusion, complement activation occurring in patients with FSGS is associated with clinical and histological severities. Low serum C3 was an independent risk factor for poor renal outcome in patients with FSGS.

## Introduction

Focal segmental glomerulosclerosis (FSGS) is a group of clinicopathological syndromes sharing a common glomerular lesion. In FSGS, podocytes are usually damaged by multiple insults directly or indirectly^1, 2^. Although several factors that lead to FSGS have been identified, approximately 80% of the cases are primary. The pathological features of FSGS are both focal and segmental, involving a minority of glomeruli and affecting a portion of the glomeruli, respectively. According to the United States Renal Data System, primary FSGS is responsible for 3.3% of all the cases of end-stage renal disease (ESRD) resulting from primary kidney disease^3^.

Previous studies have identified clinical and pathological risk factors including heavy proteinuria, reduced renal function, hypertension at the time of diagnosis, interstitial fibrosis, and glomerular sclerosis to be associated with adverse outcomes^4^. In recent years, several investigations have been undertaken to elucidate the role of permeability factors including hemopexin^5^, suPAR^6^, vascular endothelial growth factor^7^, and cardiotrophin-like cytokine-1^8^, in the pathogenesis and progression of primary FSGS. However, a consensus on the role of permeability factors in the pathogenesis or progression of FSGS is yet lacking^9^.

Previous studies have indicated that complement activation was present in FSGS patients and associated with disease progression^10, 11^. Glomerular C3 could be detectable in a small subset of patients with FSGS^12^. However, a consistent threshold of complement activation in FSGS has not been achieved. Thus, whether complement activation and low serum C3 are useful in predicting the renal outcomes is unknown. Moreover, the prevalence of low serum C3 in patients with primary FSGS has not been evaluated in any other large patient cohort. In the present study, we enrolled patients with primary FSGS and explored whether serum C3 could improve the predictive value of primary FSGS progression in order to evaluate its predictive value as a non-invasive biomarker in primary FSGS progression.

## Results

### Baseline clinical findings

A cohort of 591 patients was enrolled in the study. The male to female ratio was 1.3:1. The mean age was 38.6 ± 14.9 years. The median proteinuria was 1.43 g/24 h (IQR, 0.57–3.34), and 47.4% patients exhibited the nephrotic syndrome. The median MDRD-eGFR was 83.96 mL/min/1.73 m^2^ (IQR, 42.58–131.09).

### Differences between low and normal serum C3 patients

The baseline characteristics of the low serum C3 and the normal serum C3 groups are listed in Table 1. The disease severity parameters such as Scr (177 [82, 381]µmol/L vs. 92 [69, 149.25]µmol/L; p < 0.01) were higher in low serum C3 group than the normal C3 group, and eGFR (42.62 [17.35, 116.78]mL/min/1.73 m^2^ vs. 91.50 [51.14, 136.28]mL/min/1.73 m^2^; p < 0.01), 24 h proteinuria (1.02 [0.37, 2.79]g/24 h vs. 1.55 [0.63, 3.54]g/24 h; p < 0.05), LDL cholesterol (4.84 [4.09, 6.44]mmol/L vs. 6.21 [4.83, 9.57]mmol/L; p < 0.01), TG (1.87 [1.24, 2.76]mmol/L vs. 2.38 [1.53, 3.41]mmol/L; p < 0.01), and serum IgA (222 [162, 356]mg/dL vs. 242 [189.75, 330]mg/dL; p < 0.05) were significantly lower in low serum C3 group; however, no significant difference in Alb (30.25 ± 10.77 g/L vs. 28.53 ± 11.32 g/L; p = 0.391), SBP (128.46 ± 21.10 g/L vs. 127.77 ± 20.84 g/L; p = 0.748), DBP (82.07 ± 13.05 mmHg vs. 81.71 ± 13.26 mmHg; p = 0.790), serum IgM (158.39 ± 100.53 mg/dL vs. 188.40 ± 404.61 mg/dL; p = 0.421), and serum IgG (1032.97 ± 754.97 mg/dL vs. 950.41 ± 479.12 mg/dL; p = 0.142) was observed.

**Table 1:** Clinical characteristics of the patients at baseline according to serum C3 level.

Variables	Low C3	Normal C3	p-value
	(<85 mg/dL)	(≥85 mg/dL)	
Gender (male/female)	61/56	264/203	0.393
Age (y)	37.93 ± 14.50	39.39 ± 15.10	0.349
Scr (μmol/L)	177 (82, 381)	92 (69, 149.25)	<0.01
eGFR (mL/min/1.73 m^2^)	42.62 (17.35, 116.78)	91.50 (51.14, 136.28)	<0.01
24 hUP (g/24 h)	1.02 (0.37, 2.79)	1.55 (0.63, 3.54)	<0.05
>3.5 (number)	21	97	
<3.5 (number)	117	348	
Alb (g/L)	30.25 ± 10.77	28.53 ± 11.32	0.391
SBP (mmHg)	128.46 ± 21.10	127.77 ± 20.84	0.748
DBP (mmHg)	82.07 ± 13.05	81.71 ± 13.26	0.790
CHOL (mmol/L)	4.84 (4.09, 6.44)	6.21 (4.83, 9.57)	<0.01
TG (mmol/L)	1.87 (1.24, 2.76)	2.38 (1.53, 3.41)	<0.01
IgA (mg/dL)	222 (162, 356)	242 (189.75, 330)	<0.05
IgM (mg/dL)	158.39 ± 100.53	188.40 ± 404.61	0.421
IgG (mg/dL)	1032.97 ± 754.97	950.41 ± 479.12	0.142

Scr: serum creatinine; 24 h UP: 24 h proteinuria; Alb: serum albumin; SBP: systolic blood pressure; DBP: diastolic blood pressure; CHOL: serum cholesterol; TG: serum triglyceride.

**Figure 1:**
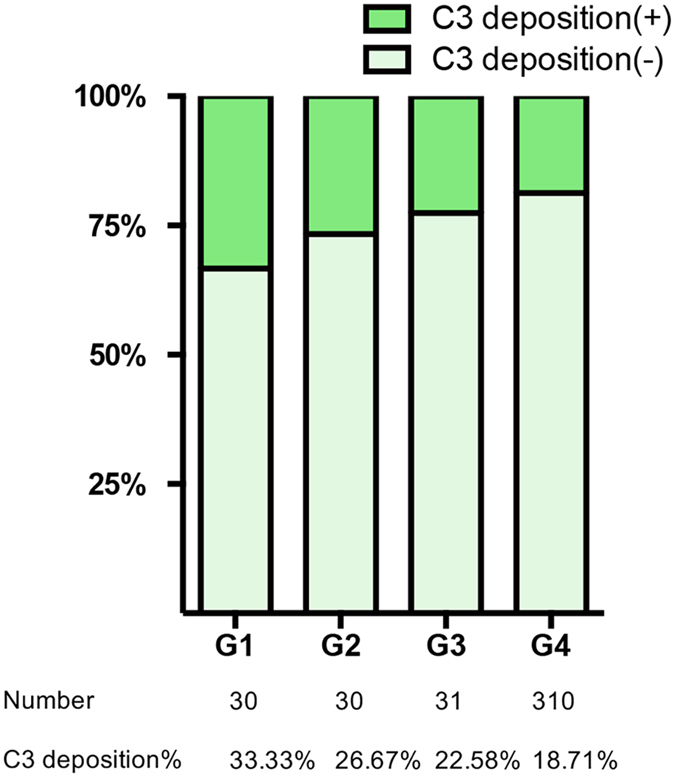
The correlation between the C3 deposition in the kidney and serum C3 levels. G1: the first third of the low serum C3 group; G2: the second third of the low serum C3 group; G3: the last third of the low serum C3 group; G4: the normal serum C3 group. The p value for trend was 0.038(χ^2^ = 4.32).

Renal biopsies were obtained. The cases with normal serum C3 displayed 11.10% [7.11, 20.00] as the median percentage of global sclerosis. 324 patients presented an interstitial injury, and 251/467 (53.7%) and 73/467 (15.6%) were scored as mild, and moderate-to-severe, respectively. Compared to the patients with normal serum C3, those with low level exhibited a significantly higher percentage of moderate-to-severe interstitial injury (28.2%, p < 0.01) (Table 2). However, no significant difference was observed in mild interstitial injury and global glomerular sclerosis. Besides, we compared the C3 deposition in the first third of the low serum C3 group(G1), the second third of the low serum C3 group(G2), the last third of the low serum C3 group(G3) and the normal serum C3 group(G4), and found a correlation between serum C3 and C3 deposition in kidney (p for trend = 0.038, χ2 = 4.32, Fig. 1).

**Table 2:** Pathological data of the patients at the time of renal biopsy.

Histological characteristics		C3 < 85 mg/dL	C3 ≥ 85 mg/dL	p-value	OR [95% CI]
		(N = 117)	(N = 467)		
TI	None	27 (23.1%)	143 (30.6%)	—	ref
	Mild	57 (48.7%)	251 (53.7%)	0.329	0.74 [0.42, 1.33]
	Moderate to severe	33 (28.2%)	73 (15.6%)	0.004	0.37 [0.20, 0.73]
	Glomerular sclerosis (%)	11.11 [6.70, 25.00]	11.10 [7.11, 20.00]	0.430	3.10 [0.96, 10]

TI: Tubulointerstitial injury.

### Study endpoints

**Figure 2:**
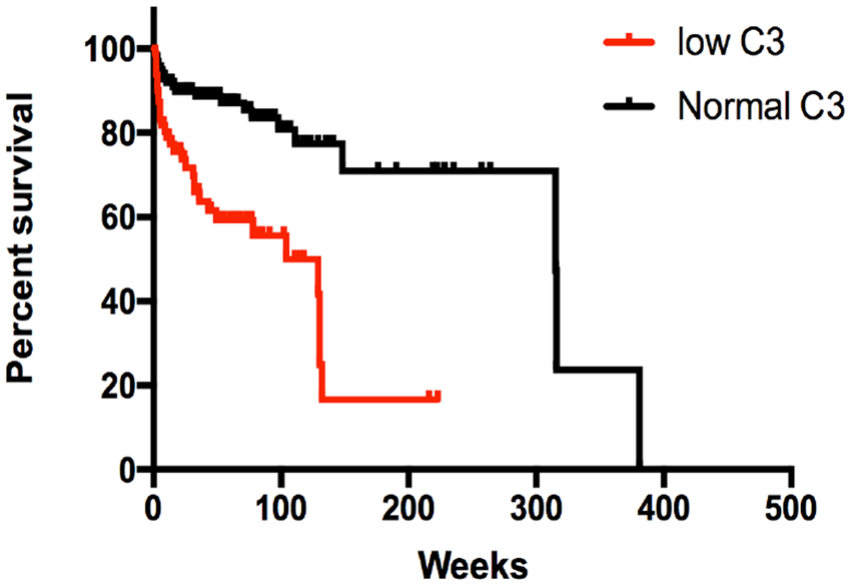
Renal survival according to serum C3 level.

The follow-up was assessed in 221 patients. During the mean follow-up of 53.3 months, 54 (24.43%) patients developed ESRD. ESRD occurred in 32 patients (37.2%) with low serum C3 as compared to 22 patients (16.3%) with normal C3 levels (p < 0.01). Kaplan–Meier analysis revealed that the median survival time of patients in normal serum C3 group was significantly longer than those in the low serum C3 group (HR = 4.044; 95% CI = 2.238–7.309; p < 0.01; Fig. 2). Table 3 presents the unadjusted and multivariable-adjusted hazard ratios (HRs) for the end point of ESRD. Univariate analysis revealed that sex, Scr, C3, SBP, DBP, and IgM among clinical data were associated with the appearance of ESRD. Only the amount of Scr (HR per 1 µmol/L increase, 1.003; 95% CI, 1.002–1.004; p < 0.001), DBP (HR per 1 mmHg, 1.027; 95% CI, 1.004–1.051; p = 0.012), and C3 (HR per 1 mg/dL increase, 0.984; 95% CI, 0.970–0.999; p = 0.035) remained as significant predictors of ESRD as proposed by multivariate analysis. In ROC curve analysis, serum C3 levels had a significant predictive value for the renal outcome (AUC = 0.650, p = 0.001).

**Table 3:** Univariate and multivariate analyses of clinical manifestations in Cox proportional hazards model.

Parameter	Univariate		Multivariate	
	p-value	HR [95% CI]	p-value	HR [95% CI]
Gender (male/female)	0.033	0.526 [0.292–0.949]	0.314	0.75 [0.405–1.288]
Age (y)	0.956	1.01 [0.982–1.019]		Not selected
Scr (µmol/L)	<0.001	1.003 [1.002–1.004]	<0.001	1.003 [1.002–1.004]
Serum C3 (mg/dL)	<0.001	0.978 [0.966–0.990]	0.035	0.984 [0.970–0.999]
Serum C4 (mg/dL)	0.783	0.999 [0.994–1.005]		Not selected
24 h UP (g/24 h)	0.860	0.993 [0.917–1.075]		Not selected
Alb (g/L)	0.480	0.99 [0.964–1.017]		Not selected
SBP (mmHg)	<0.001	1.021 [1.009–1.033]	0.982	1.000 [0.984–1.016]
DBP (mmHg)	0.030	1.021 [1.002–1.041]	0.012	1.027 [1.004–1.051]
CHOL (mmol/L)	0.096	0.894 [0.782–1.020]		Not selected
TG (mmol/L	0.485	0.930 [0.757–1.141]		Not selected
IgM (mg/dL)	0.012	0.994 [0.990–0.999]	0.627	0.999 [0.994–1.003]
IgA (mg/dL)	0.556	1.000 [0.999–1.001]		Not selected
IgG (mg/dL)	0.057	1.000 [1.000–1.001]		Not selected

Scr: serum creatinine; 24 h UP: 24-hour proteinuria; Alb: serum albumin; SBP: systolic blood pressure; DBP: diastolic blood pressure; CHOL: serum cholesterol; TG: serum triglyceride.

### Correlation between serum C3 level and activation of complement system

**Figure 3:**
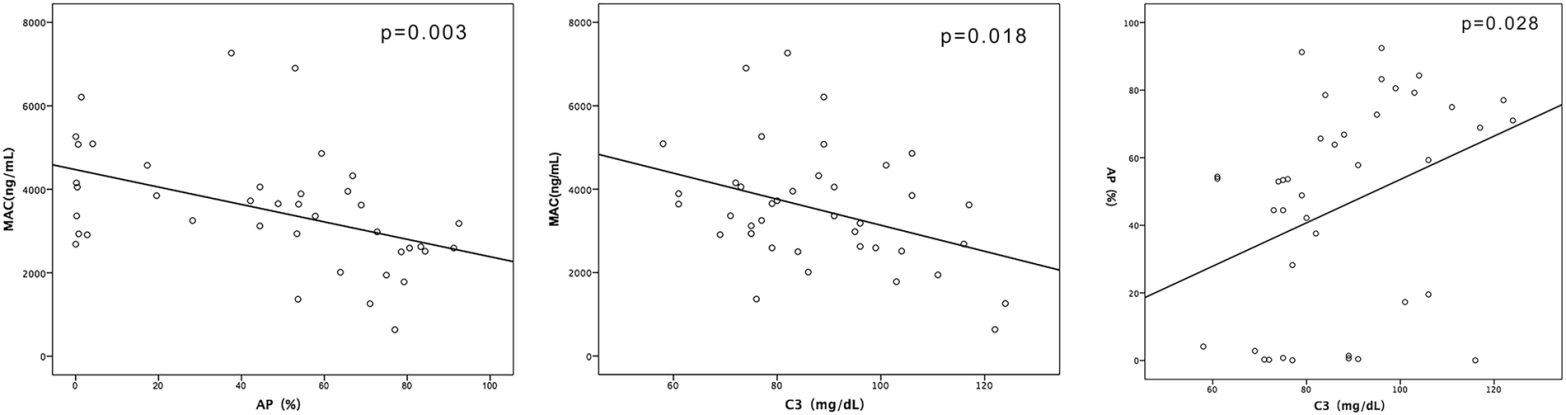
Correlation between serum C3 level, alternative pathway activation, and complement system activation in 40 FSGS patients.

To further elaborate the cause for low serum C3 and poor outcome of FSGS, we included 40 patients with primary FSGS and examined the serum alternative pathway activation and serum MAC level. A significant correlation was established between MAC and AP (p = 0.003, r = −0.459), MAC and serum C3 (p = 0.018, r = −0.371), and AP and serum C3 (p = 0.028, r = 0.347) (Fig. 3). Next, we compared the activation of the complement system in patients with different tubulointerstitial injuries. The result revealed that as compared to patients with none-to-mild TI, those with moderate-to-severe TI were predisposed towards a lower level of serum C3 (88.38 ± 18.899 vs. 87.38 ± 15.794 mg/dL), lower level of AP (44.57 ± 32.71 vs. 46.50 ± 30.85%), and a higher level of serum MAC (3646.72 ± 1573.09vs. 431.02 ± 1334.34 ng/dL).

## Discussion

In this study, for the first time, we found that low serum C3 was an independent risk factor for the development of ESRD in FSGS. As shown in Fig. 2, renal survival was significantly higher in patients with normal serum C3 as compared to those with low serum C3. Thus, it can be hypothesized that the activation of the serum complement system may play a crucial role in the pathogenesis and outcome of FSGS.

FSGS is viewed as a group of clinicopathological syndromes with a common glomerular lesion and mediated by multiple insults causing podocyte injury^13^. Although the cause of FSGS is heterogeneous, the podocyte injury was the core of FSGS pathogenesis^14^. Circulating factors, including cardiotrophin-like cytokine-1 and soluble urokinase plasminogen activator receptor, were recognized as the leading causes for primary FSGS^15^. However, whether FSGS is an immune-mediated disease is yet uncertain, although the initial treatments for primary FSGS consist of steroid and immunosuppressive drugs. Furthermore, studies have also indicated that the efficacy of some of these drugs may be attributed to non-immune effects. Moreover, a recent study showed that the immune-mediated disease might associate with FSGS in basic research^16^.

Over the past decade, predictors of renal failure in FSGS have been assessed in several clinicopathological studies. Clinical and laboratory features, including the degree of proteinuria, decreased renal function, the presence of hypertension, the absence of macroscopic hematuria, have been found to be independently associated with the progressive renal disease. In addition, genetic alterations caused FSGS^17, 18^. The histological features, such as collapsing type glomerulosclerosis, and interstitial fibrosis, were identified as independent predictors of renal failure; however, the role of complement activation in FSGS was not described. Thus, clarifying the role of complement activation in FSGS was essential.

The complement system functions as a part of the innate immune system, and the activation of complement pathways exerts a deleterious effect on kidneys. Recent studies found that the over-activation of complement was associated with the pathogenesis of glomerular and tubulointerstitial injury. Complement can be activated by classical, alternative, and lectin pathways. The nephritogenic effects of complement activation are mediated by inflammation, the release of chemotactic factors, and the generation of membrane attack complex C5b-9. The level of serum C3 indicates the activation of complement.

Recent studies focusing on the complement activation have provided new insights into the pathogenesis of IgA nephropathy^19, 20^, lupus nephritis, secondary FSGS, C3 nephropathy^21^, and HUS^22^. In secondary FSGS^23^, the complement alternative pathway activation was associated with features of capillary wall injury. Sethi *et al*.^24^ used mass spectrometry to analyze the glomeruli isolated by laser-capture microdissection and showed that they contain the components of the alternative pathway of complement activation and the terminal pathway C5–C9, respectively. A genome-wide association study of IgA nephropathy identified three independent loci in the major histocompatibility complex (MHC) and a common deletion in CFHR1 and CFHR3 that reached genome-wide significance, emphasizing the crucial role of complement activation in the disease^25^.

Our large group study showed that low serum C3 might play a vital role in the pathogenesis of FSGS. ELISA results support that serum C3 was an indicator of serum complement activation. The complement activation in FSGS has been indicated as crucial by several studies. One study measured the complement fragments Ba, Bb, C4a, and sC5b-9 in plasma and urine in 19 patients with FSGS and found that the complement system was activated in patients with primary FSGS, and elevated levels of plasma Ba correlated with a severe disease^12^. Zhao *et al*. demonstrated that glomerular deposition of IgM and C3 were unfavorable therapeutic responses resulting in poor renal outcomes in primary FSGS^26^. Lenderink *et al*. proved that C3 was present as an activation fragment and colocalized with glomerular IgM, suggesting that glomerular IgM may harbor a cognate ligand^11^. All of these suggest that complement activation plays a role in the outcome of FSGS. The present study showed that serum C3, an indicator of complement activation and a readily available biochemical index, could be an indicator of the outcome of FSGS. Nevertheless, the cause of activation of complement in these patients is yet unclear necessitating further research.

Moreover, we found that as compared to patients with normal serum C3, patients with low serum C3 had a significantly higher percentage of interstitial injury. And C3 deposition in kidney are more often observed in low serum C3 group. In the group of 40 patients, those with moderate-to-severe interstitial injury are predisposed towards excessive complement activation by the alternative pathway. The tubulointerstitial fibrosis was recognized as a significant predictor of ESRD in patients with FSGS^27^. Previous studies indicated that C3 deposition associated with interstitial injury^28, 29^. In the experimental focal segmental glomerulosclerosis, the intratubular formation of C5b-9 is known as a specific promoter of peritubular myofibroblast accumulation^30^. Therefore, the activation of complement could also cause tubulointerstitial injury, leading to fibrosis in primary FSGS.

Our study harbors the inherent limitations of a retrospective observational investigation. First, the relationship between serum C3 level and progression of renal damage must be interpreted cautiously regarding association rather than causality. Second, the complement components including activated C3a, C4–C3 complexes, or soluble C5b-9 were not detected in the serum and urine in the larger group. Also, we did not perform the additional staining for complementary components in glomeruli, which were previously suggested to be putative predictors of disease activity as alternatives to C3 in the other kidney diseases. Third, the serum C3 levels were measured only during the renal biopsy. Thus, whether low serum C3 persisted throughout the disease course is unknown. Therefore, monitoring the serum C3 level to further clarify the clinical implications would be valuable. Moreover, we could neither deduce the reason for the activation of complement system nor the role of different complement pathways in FSGS.

In conclusion, our study confirmed that the serum C3 level is an independent risk factor for renal survival in FSGS, and the serum C3 level could be utilized for the prediction of prognostic value. These findings are in agreement with the potential theory that complement activation is involved in the progression of FSGS.

## Methods

### Design

This study was approved by the Ethics Committee of the Ruijin Hospital, Shanghai Jiao Tong University School of Medicine. The methods were carried out in accordance with the relevant guidelines. Patients’ information was managed according to applicable data protection regulations. All subjects provided written informed consent.

### Patients

The patients enrolled in this study were selected based on the diagnosis of FSGS in Shanghai Jiao Tong University Affiliated Ruijin Hospital between January 2008 and December 2012. Patients with Henoch-Schönlein purpura, liver diseases, diabetes, systemic diseases, and any type of secondary FSGS were excluded. Patients with Hepatitis C or B infection were also excluded. The date of renal biopsy was established as the baseline point for each patient. The diagnosis of FSGS was based on the histological assessment of renal biopsy tissue with hematoxylin and eosin, Masson’s trichrome, periodic acid-Schiff, and methenamine silver for light microscopy and staining with antibodies against IgG, IgA, IgM, C1q, and C3 for immunofluorescence. Tubulointerstitial injury was scored as none, mild(<25%), moderate(25–50%) and severe(>50%) based on the severity and extent of tubular atrophy, interstitial inflammatory cell infiltration, and interstitial fibrosis^31, 32^.

The medical records were reviewed, and the following information was recorded at the time of the renal biopsy: patient age, sex, Hb, SBP, DBP, CHOL, TG, serum IgA, serum IgG, serum IgM, 24 h urine protein excretion, and serum creatinine level. The mean values of the two latest estimations within the month before the renal biopsy were selected. We calculated the eGFR in adults using the abbreviated Modification of Diet in Renal Disease study equation^33^: eGFR (mL/min/1.73 m^2^) = 186 × (serum creatinine in mg/dl^−1.154^) × (age^−0.203^) × (0.742 if female) × (1.21 if black). The Schwartz formula was used to calculate the eGFR in children.

### Serological analysis

Serum concentrations of MAC were measured by commercially available sandwich enzyme-linked immunosorbent assay (ELISA) Kit (SC5b-9 Plus EIA Kit, Quidel, San Diego, CA, USA). Alternative pathway activity was measured using the Wieslab Complement AP Assay (Complement system Alternative Pathway Wieslab^®^, Euro-Diagnostica AB, Lund, Sweden). The absorbance at 450 nm (MAC) or 405 nm (alternative pathway activity) was determined using a microplate reader (BioTek Instruments, Inc., VT, USA). The alternative pathway activity was calculated as follows: (sample-negative control)/(positive control-negative control) × 100.

### Study endpoints

The date of renal biopsy of each patient was established as the baseline point. The follow-up time was considered as the interval between the biopsy and the last visit of the outpatient, death, or ESRD (defined as the onset of chronic dialysis or renal transplantation). The primary endpoint was defined as the cumulative percentage of patients who developed ESRD during the study (renal survival). None of the patients succumbed to mortality before the ESRD.

### Statistical analyses

The results were reported as the mean ± SD when normally distributed or as the median (interquartile range [IQR]) in the other cases. The comparisons of continuous variables between the two groups were assessed using the unpaired t-test or the Mann-Whitney U test as appropriate. The differences in the proportions of different patient groups were compared by the Fisher’s exact test. A p-value < 0.05 was considered to be statistically significant.

Kaplan–Meier and Cox proportional hazards analyses evaluated the effect of serum C3 on renal survival. The primary endpoint was ESRD. Univariate survival was compared using the log-rank test. All univariate tests were two-sided, with an α level of 0.05. The Cox proportional hazards model was used to estimate the adjusted relative risk of each parameter with respect to renal survival. The survival time for each patient was computed from the baseline evaluation to the last follow-up or the primary endpoint, ESRD. The variables that were previously found affecting the renal survival were included in the Cox proportional hazards model. Thus, the variables were selected by backward elimination using the likelihood ratio tests. Calculations were performed using SPSS statistical software (version 19.0; SPSS, Chicago, IL, USA).
